# First-in-Center Experience with a Novel Intravascular Lithotripsy System: The Shunmei ShockFast™ Intravascular Lithotripsy System Device for the Treatment of Severe Calcified Coronary De Novo Lesions

**DOI:** 10.3390/life16030426

**Published:** 2026-03-05

**Authors:** Giacomo Maria Cioffi, Julius Jonas Jelisejevas, Ioannis Skalidis, Peter Wenaweser, Pascal Meier, Mario Togni, Stéphane Cook

**Affiliations:** Department of Cardiology, University and Hospital Fribourg, 1708 Fribourg, Switzerland

**Keywords:** coronary artery disease, severe coronary calcifications, intravascular lithotripsy, optical coherence tomography

## Abstract

**Background:** Intravascular lithotripsy (IVL) has emerged as a safe and effective modality for treating severely calcified coronary lesions. While the Shockwave™ system is well-established, clinical data on newer IVL platforms such as the Shunmei ShockFast™ system remain limited. **Objectives:** To evaluate the safety, feasibility, and procedural outcomes of the ShockFast IVL device in patients with heavily calcified de novo coronary artery disease. **Methods:** We conducted a prospective, single-center case series of 16 patients undergoing percutaneous coronary intervention (PCI) with the ShockFast IVL system between June and December 2025. Inclusion required angiographic or optical coherence tomography (OCT) evidence of severe coronary calcification. The primary efficacy endpoint was acute procedural success and absence of in-hospital MACE. Secondary endpoints included, among others, device deliverability, presence of calcium fracture and post-stent expansion metrics. **Results:** All patients underwent successful lithotripsy delivery with the ShockFast IVL system. Acute procedural success was 100%, with no intraprocedural complications, abrupt closure, or in-hospital MACE. OCT was performed in 50% of cases and demonstrated calcium fractures in all imaged lesions, with ≥2 fractures in 63% of cases. Median stent expansion was 90% [IQR 9], with no major malapposition or edge dissections. Quantitative coronary analysis showed a median acute lumen gain of 1.86 mm [0.62]. **Conclusions:** The ShockFast IVL system showed excellent safety and procedural performance in this first-in-center experience. Outcomes were encouraging and consistent with those reported in early-stage studies of other IVL platforms. These findings support the clinical feasibility of ShockFast as a novel tool for calcium modification in complex PCI.

## 1. Introduction

Severe coronary artery calcification remains one of the most challenging entities of percutaneous coronary intervention (PCI), with an increased risk of stent failure, higher rates of periprocedural complications, and worse long-term outcomes [1,2,3,4,5].

The prevalence of coronary calcification increases with age—for example, over 90% of men and 70% of women above 70 years old have coronary calcific plaque [6,7]. These calcified lesions often necessitate specialized plaque modification techniques and devices to enable adequate lesion preparation and stent deployment. Traditionally, operators have relied on non-compliant balloons and/or atherectomy devices to modify calcium [8,9,10,11]. While effective in many cases, these conventional technologies have limitations, including the risk of significant periprocedural complications (such as embolization, severe dissections, or perforations) [8,12]. This has driven the search for alternative, safer calcium modification strategies in PCI [13].

Among them, intravascular lithotripsy (IVL) was first introduced for coronary use around 2017–2018 (following CE-mark approval in 2017) and adapts the principles of extracorporeal shockwave lithotripsy (used to break kidney stones) for intracoronary use [14]. Instead of ablating or cutting plaque, IVL delivers pulsatile acoustic pressure waves via a specialized balloon catheter to selectively fracture calcified plaque in situ, while leaving surrounding soft tissue largely unharmed.

The Shockwave™ C2 coronary IVL system (Shockwave Medical, Santa Clara, CA, USA) was the first such device that demonstrated a high rate of lesion modification success with an excellent safety profile in clinical studies [15,16]. Mechanistically, intracoronary imaging has confirmed that IVL produces multiple calcium fractures within the plaque, which facilitate greater lumen expansion and stent deployment. Beyond controlled trials, real-world registries have corroborated these findings: IVL is consistently associated with high success rates and low complication rates across various patient subsets [17,18,19,20,21,22]. Since its introduction in 2021, the adoption of coronary IVL has grown rapidly, at times even exceeding the use of atherectomy in contemporary practice, underlining the perceived ease of use and safety by operators.

Encouraged by the success of Shockwave™ IVL, new lithotripsy catheters have been developed. One such device is the ShockFast™ coronary IVL system (Shunmei Medical, Shenzhen, China), which received regulatory approval in some countries and has shown promising initial results, but published clinical data currently remain extremely limited. Therefore, we present our first clinical experience using the Shunmei ShockFast™ IVL catheter for severe calcified de novo coronary lesions.

## 2. Material and Methods

### 2.1. Study Population

Consecutive patients presenting between June 2025 and December 2025 with acute or chronic coronary syndromes and severely calcified de novo coronary artery lesions undergoing PCI were prospectively included at a tertiary Swiss center. This prospective study stems from the Cardio-FR registry (NCT04185285), a registry initiated in January 2015 at the University & Hospital of Fribourg (Switzerland) and still prospectively ongoing. The registry is conducted in accordance with the Declaration of Helsinki, was approved by the Institutional Review Board, and received approval from the local ethics committee (003-REP-CER-FR). All patients provided written informed consent.

### 2.2. Study Eligibility Criteria

The *angiographic inclusion criteria* were as follows: at least one stenosis of >50% of diameter stenosis, with ≤50 mm of lesion length, distal reference vessel diameter of 2.0 to 4.0 mm by visual estimation, and presence of severe calcifications. Severe calcification was classified according to the modified ACC/AHA angiographic classification for coronary artery calcification [23,24,25].

The *OCT inclusion criteria* were defined as the presence of a revised or original OCT calcium score of more than 3 points [26,27].

*Exclusion criteria* included severe impaired acute or chronic renal disease, cardiogenic shock, severe chronic obstructive pulmonary disease, and allergy to the contrast agent.

### 2.3. Study Device

**Figure 1:**
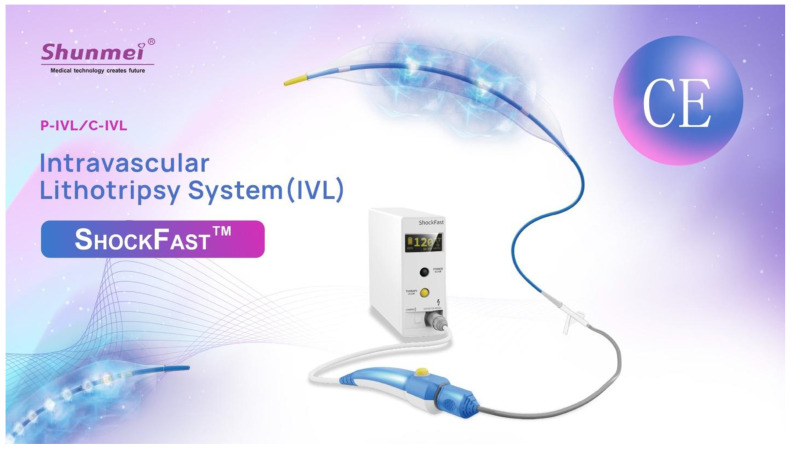
The Shunmei ShockFast™ IVL system.

The ShockFast™ (Shunmei Medical, Shenzhen, China) coronary IVL system and coronary IVL catheter received the CE marking on 18 May 2025. It consists of a 0.014-inch guidewire-compatible, contrast fluid-filled balloon angioplasty catheter with 2 lithotripsy emitters incorporated into the shaft of either a 12 mm or 15 mm long balloon segment (Figure 1). The coronary IVL system is delivered on a rapid exchange catheter and is available in 2.5, 2.75, 3.0, 3.25, 3.5, and 4.0 mm diameters. Each catheter is intended for a single use and can provide up to 120 total pulses delivered in 10 pulses per cycle, singularly or in total, by double-pressing the delivery button. Balloon treatment pressure and nominal pressure are 4 and 6 atm, respectively, with a rated burst pressure of 10 atm.

### 2.4. Study Procedure

Patients that provided written informed consent and met study eligibility criteria were included. Intracoronary imaging by OCT was highly encouraged but not mandatory and was ultimately at operator’s discretion. When performed, at least 3 time points were requested: at baseline (pre-IVL treatment), directly post-IVL treatment, and following stent deployment and its optimization. Additional pullbacks between additional plaque modification or tools were encouraged. The IVL catheter was planned as the upfront treatment and delivered over a 0.014-inch guidewire of choice. If the lesion was deemed uncrossable or if the IVL catheter could not be advanced, adjunctive treatment (e.g., buddy wire, guiding catheter extension, pre-dilatation, or atherectomy) was performed at operator’s discretion before reinsertion of the IVL catheter.

A 1:1 sized—to distal reference vessel diameter (RVD)—IVL balloon was inflated to 4–6 atm with delivery of all pulses to the target lesion. The IVL treatment was repeated until full IVL balloon expansion was achieved or until all pulses were depleted. If there was still persistent incomplete lesion preparation despite maximum number of pulses (i.e., residual stenosis >50%), an additional IVL catheter was used. Additional non-compliant balloon (NCB) dilatation was performed after IVL treatment and prior to stent implantation to improve lumen gain. Dual antiplatelet therapy (DAPT) was prescribed as per current guidelines and accordingly with the patient’s clinical presentation [28,29].

### 2.5. Data Analysis and Data Management

All angiographic imaging data were analyzed offline via the Philips Azurion (Horgen, Switzerland) intervention suite integrated quantitative coronary analysis (QCA) software, version 1.2.3. OCT images were analyzed offline via the Ultreon™ 2.0 OPTIS Software (Abbott Vascular Inc., Santa Clara, CA, USA). Source data were collected offline by local investigators and subsequently transferred into an electronic data capture system for statistical analysis.

### 2.6. OCT Image Analysis

OCT image analysis was performed offline using the OPTIS Software (Abbott Vascular Inc., Santa Clara, CA, USA). If images were not already correctly calibrated, they were recalibrated using the dedicated adjustment tool. For each case, we collected the following quantitative measurements: minimum lumen area (MLA) (mm^2^), minimum lumen diameter (MLD) (mm), proximal and distal reference mean diameter (mm), proximal and distal external elastic lamina (EEL) mean diameter (mm) measured at 5 mm proximally and distally to the lesion length, and lesion length (mm). Calcium arc (degrees), calcium thickness (mm), and calcium length (mm) were measured at the maximum calcium site. Lumen gain after IVL treatment (mm^2^), final minimal stent area (MSA) (mm^2^), final acute lumen gain (mm^2^)—defined as an increase in lumen area between initial MLA and final lumen area at MSA—and final stent expansion (SE) (%) were also measured. Additionally, we collected the following qualitative characteristics: presence of calcified nodule, type of calcified nodule—eruptive or non-eruptive—revised OCT calcium score [27], original OCT calcium score [26], presence of calcium fractures after IVL treatment at maximal calcium site, presence of proximal or distal medial edge dissections, and presence of minor or major (>400 um for a length of more than 1 mm) malapposition [30,31].

### 2.7. Endpoints

The primary safety endpoint of this study was the incidence of in-hospital major adverse cardiovascular events (MACE), defined as a composite of cardiac death, target vessel myocardial infarction (TV-MI)—defined as acute myocardial necrosis occurring within the territory of a previously treated vessel with an elevation of CK-MB > 10× the upper limit of normality or > 5× with the presence of new Q-waves [32]—TIA/stroke, and post-procedural type 4a MI—defined as a PCI-related MI occurring within 48 h of the procedure, with a high-sensitivity cardiac troponin (hs-Tn) elevation > 5 times the upper reference limit (URL) [33]. Device-related complications such as coronary perforation, flow-limiting dissection, abrupt vessel closure, or sustained ventricular arrhythmias were also monitored.

The primary efficacy endpoint was acute procedural success, defined as achievement of angiographic success of the treating ShockFast™ IVL catheter device with a residual diameter stenosis < 30% by QCA and absence of in-hospital MACE.

Secondary endpoints included device success—defined as successful delivery, activation, and completion of lithotripsy cycles at the target lesion—and several OCT metrics, among which are the presence of calcium fractures and stent expansion of >80%.

No formal hypothesis testing was performed, as this was an observational, exploratory case series intended to assess the initial feasibility, safety, and procedural characteristics of the ShockFast™ IVL system in a real-world coronary population.

### 2.8. Statistical Analysis

Categorical variables are displayed as absolute numbers and percentages. Continuous variables are presented as means (±standard deviation) or medians (interquartile ranges [IQRs]), as appropriate. All the analyses were conducted using STATA version 17 (College Station, TX, USA).

## 3. Results

In total, we included 16 patients that presented with severely calcified lesions as assessed by angiographic or OCT means and were treated with the ShockFast™ IVL. Of these, five (31%) were females, only four (25%) presented with diabetes mellitus, half of them had a previous PCI, and 12 (75%) presented with a chronic coronary syndrome. Four (25%) patients presented with acute coronary syndromes, with either STEMI or NSTEMI/UA as the clinical presentation. Further additional clinical characteristics are shown in Table 1.

The main treated vessel was the RCA (63%), and most patients (88%) presented with a lesion classified as type C according to the AHA/ACC angiographic calcium classification. We observed no balloon-uncrossable lesions, and no atherectomy was performed. Pre-dilatation before ShockFast™ IVL usage was performed in 12 (75%) patients. The median maximal diameter of the ShockFast™ IVL balloon was 2.5 mm [IQR 1], and no additional pre-dilatation or lesion preparation was performed after the IVL use. DES implantation followed in 14 (88%) patients, whereas two (12%) were treated with a drug-coated balloon (DCB)-only strategy. Post-dilatation of the implanted stent with an NCB followed in all patients. We observed no abrupt vessel closure, no-reflow phenomenon, dissections, or perforations, with an acute procedural success and device success of 100% for both. Additional angiographic characteristics and periprocedural outcomes are displayed in Table 2 and Table 3, respectively.

For the QCA, the median length of the 16 treated lesions was 28.9 mm [11.8], with a median reference vessel diameter of 2.5 mm [0.46] and a median MLD of 1.21 mm [1.06]. The pre-procedural percentage diameter stenosis (%DS) was 89.5% [IQR 17] with an important improvement after ShockFast™ IVL treatment with a residual %DS of 60.5% [IQR 32]. The final MLD after DES implantation and post-dilatation reached a median 3.1 mm [0.53] with a median 1.86 mm [0.62] acute lumen gain. Further QCA characteristics are presented in Table 4. QCA results were consistent even after stratification based on the method of assessment (angiography-based only vs. OCT-based)—Table 5—with only a significant difference in residual diameter stenosis directly after IVL treatment (41.88 ± 20.92 in the angiography group versus 66.25 ± 14.08 in the OCT-based group, with a *p*-value of 0.016).

**Figure 2:**
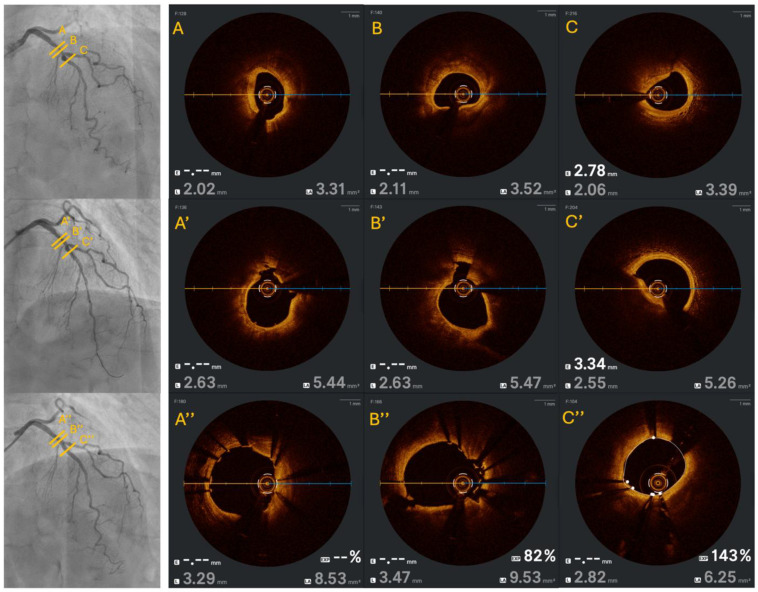
**OCT case example of treatment with the Shunmei ShockFast™ IVL system.** (**A**) Initial MLA before IVL treatment. Presence of a concentric mixed deep and superficial thick calcification. (**B**) Cross section near the MLA, with presence of diffuse circumferential, deep and superficial, thick calcium. (**C**) Presence of an eccentric, deep calcification. (**A’**) MLA directly after ShockFast™ IVL treatment, with presence of lumen gain (from 3.31 mm^2^ to 5.44 mm^2^) and two calcium fractures (12 o’clock and 3 o’clock, respectively). (**B’**) Same cross section as 2B, with evidence of a deep, large, calcium fracture and significant lumen increase. (**C’**) Eccentric deep calcification after ShockFast™ IVL treatment; although eventual fracture assessment is limited, there is the clear presence of a dissection (9 o’clock) and lumen gain. (**A’’**,**B’’**) Final OCT run after stent implantation and optimization. The stent is greatly apposed and expanded, with evidence of stretched calcium fractures and important lumen gain. (**C’’**) Although the deep eccentric calcification was not clearly fractured, there is great lumen gain.

OCT was performed in half of the patients and confirmed the severity of the treated calcified lesions, with a median revised OCT calcium score of 3 [0] and a median original OCT calcium score of 4 [0]. Notably, eruptive calcified nodules were present in three (38%) patients. Fractures were shown at the site of maximum calcification in every OCT run after ShockFast™ IVL treatment, with ≥2 fractures present in five (63%) patients (Figure 2).

Results assessed by OCT means showed a median final MSA of 4.97 mm^2^ [3.53] and a median SE of 90% [IQR 9]. Furthermore, no major malapposition, medial proximal, or distal edge dissections were noted. Additional OCT data is presented in Table 6.

Peri-procedurally, we observed no in-hospital MACE, with no post-procedural myocardial infarction (type 4a) or TV-MI, as further confirmed by the absence of dynamic changes in cardiac biomarkers (pre-procedural CK-MB at 16 U/L [IQR 6] and post-procedural CK-MB at 15 [IQR 9]), as seen in Table 7.

## 4. Discussion

In this first-in-center experience, we report the safety and procedural feasibility of the Shunmei ShockFast™ intravascular lithotripsy (IVL) system in a series of patients presenting heavily calcified de novo coronary artery lesions. Our findings suggest that this novel IVL system achieves good lesion modification and stent optimization with an apparently great procedural safety profile, mirroring outcomes previously reported with the established Shockwave™ IVL technology [21,22].

IVL has established itself as an effective and user-friendly alternative to atherectomy, conventional, or specialized balloon strategies, offering selective calcium fracture with minimal soft tissue trauma. Since its introduction, Shockwave™ IVL has demonstrated high procedural success and low complication rates in both controlled trials and real-world registries and has become a routine tool in the treatment of calcified lesions [12,13,14,20,21,34,35,36,37,38,39,40]. The introduction of new IVL platforms such as ShockFast™ aims to further expand the therapeutic landscape.

Our prospective case series included 16 patients treated with ShockFast™ IVL for severely calcified lesions, of whom the majority had chronic coronary syndromes and complex type C lesions. Despite lesion complexity, we achieved 100% device and procedural success, with no observed cases of perforation, flow-limiting dissection, no-reflow, or in-hospital MACE. These findings are within the reported range of the safety profile of the established Shockwave™ IVL, where serious complications such as perforations have been reported in <0.5% of cases in multicenter trials like DISRUPT CAD III and IV [21,22,37,40]. Importantly, no lesion required additional atherectomy, and even in tight lesions, balloon deliverability was high, with pre-dilation needed in three-quarters of cases.

Intravascular imaging, when performed, confirmed the hallmark features of effective IVL therapy [41,42,43]. OCT analysis showed post-lithotripsy calcium fractures in all imaged lesions, with ≥2 fractures observed in over 60% of cases within the range of the published OCT sub-studies of Shockwave™ IVL, where 79–82% of lesions demonstrated calcium disruption. Moreover, acute lumen gain, final minimal stent area, and stent expansion were optimal, with a median final stent expansion of 90% and absence of major malapposition or edge dissection. These results underscore ShockFast™’s ability to generate sufficient radial force to fracture both superficial and deep calcium, improving vessel compliance and optimizing final stent geometry.

Procedural efficacy, measured by angiographic residual stenosis and/or—when present—OCT assessment, was consistent with expectations for effective plaque modification. The QCA post-stenting minimum lumen diameter improved substantially, and acute gain exceeded 1.8 mm. The degree of lumen gain achieved with ShockFast™ is within the range of outcomes reported with Shockwave™ in studies such as DISRUPT CAD II and III, where high procedural success (≥92%) and favorable angiographic and imaging results confirmed IVL’s effectiveness in modifying calcified plaque [20,21,37,44].

Our findings also highlight the potential utility of ShockFast™ in diverse lesion and patient subsets, including long lesions and complex vessel anatomies. The deliverability of the balloon was consistently high, and in no case was the lesion crossing unsuccessful. Finally, the highly competitive cost of the device compared to the competitors could be of potentially high interest.

Head-to-head comparative studies between ShockFast™ and Shockwave™ IVL systems—ideally incorporating core lab-adjudicated imaging endpoints and clinical follow-up—will be critical to further define their relative performances, particularly in high-risk anatomical subsets such as left main or bifurcation disease. Economic analyses may also help determine the value proposition of alternative IVL systems in cost-conscious healthcare environments.

## 5. Limitations

Our study must be interpreted within the context of several limitations. First, this study presents a very small sample size and lacks a control group, which restrict definitive conclusions regarding comparative effectiveness. Additionally, the absence of complications within this small cohort does not establish a definitive safety profile. Second, there is the presence of potential type II errors in the safety assessment. Third, the study focused on acute procedural success, strategy success, and in-hospital outcomes, with the absence of mid- and long-term clinical follow-up. Clinical follow-up at 30 days and 1 year is planned as part of the ongoing registry and will be reported separately. Fourth, comparisons between the different IVL devices were indirect and hypothesis-generating only. Fifth, although strongly advised, intracoronary imaging was operator-dependent and only performed in half of the presented patients. This limited full mechanistic assessment of the calcium fracture and stent expansion across the cohort and might have introduced selection bias. Additionally, independent core lab adjudication was not performed.

## 6. Conclusions

In this first-in-center clinical experience, the new ShockFast™ coronary IVL system appeared to be effective and safe, with great deliverability and performance in the treatment of severely calcified de novo coronary lesions. The device consistently achieved lesion modification, stent expansion, and procedural success without complications. These results are encouraging and consistent with those reported in early-stage studies of other IVL platforms. These findings support the integration of ShockFast™ into the calcium modification toolkit for complex PCI, but larger studies with longer-term follow-up and head-to-head comparison with the established Shockwave™ IVL system are warranted to confirm ShockFast™’s clinical durability and further clarify its role in contemporary practice.

## Figures and Tables

**Table 1:** Clinical characteristics.

	*N* of Patients = 16
Age, *n* (±SD)	76.4 ± 7
Female, *n* (%)	5 (31)
BMI, kg/m^2^ [IQR]	28 [11.4]
Diabetes mellitus, *n* (%)	4 (25)
Dyslipidemia, *n* (%)	15 (94)
Arterial hypertension, *n* (%)	14 (88)
Active smoking, *n* (%)	4 (25)
Family history for CAD, *n* (%)	3 (19)
Chronic alcohol consumption, *n* (%)	2 (13)
Previous MI, *n* (%)	2 (13)
Previous PCI, *n* (%)	8 (50)
Previous CABG, *n* (%)	1 (6)
Clinical presentation, *n* (%)	
STEMI	1 (6)
NSTEMI/UA	3 (19)
CCS	12 (75)
LVEF, % [IQR]	60 [17]

Data are mean (SD = standard deviation), median (IQR = interquartile range), or number (*n* = number; % = percentage), as appropriate. BMI = body mass index; CAD = coronary artery disease; MI = myocardial infarction; PCI = percutaneous coronary intervention; CABG = coronary artery bypass grafting; STEMI = ST elevation myocardial infarction; NSTEMI = non-ST elevation myocardial infarction; UA = unstable angina; CCS = chronic coronary syndrome; and LVEF = left ventricular ejection fraction.

**Table 2:** Angiographic characteristics.

	*N* of Patients = 16
Number of vessels treated per procedure, *n* [IQR]	1 [0]
Vessel treated, *n* (%)	
RCA, *n* (%)	10 (63)
LAD, *n* (%)	6 (37)
ACC/AHA coronary lesion calcification	
Severe calcification, *n* (%)	16 (100)
Type B2, *n* (%)	3 (19)
Type C, *n* (%)	13 (81)
DES implantation, *n* (%)	14 (88)
Number of DES implanted, *n* [IQR]	2 [2]
Maximum DES diameter, mm [IQR]	3 [4]
DES total length, mm (±SD)	43 ± 27
DES implantation pressure, atm (±SD)	12 ± 6
DCB use, *n* (%)	2 (13)
Pre-dilation before IVL	
SC balloon, *n* (%)	5 (31)
SC balloon max. diameter, mm [IQR]	1.5 [1]
SC balloon max. pressure, atm [IQR]	14 [4]
NC balloon, *n* (%)	12 (75)
NC balloon max. diameter, mm [IQR]	2.5 [0.375]
NC balloon max. pressure, atm [IQR]	16 [7]
ShockFast™, *n* (%)	16 (100)
ShockFast™ max. diameter, mm [IQR]	2.5 [1]
ShockFast™ max. pressure, atm [IQR]	6 [0]
ShockFast™ pulses, pulses [IQR]	120 [35]
Post-dilatation, *n* (%)	16 (100)

Data are mean (SD = standard deviation), median (IQR = interquartile range), or number (*n* = number; % = percentage), as appropriate. RCA = right coronary artery disease; LAD = left anterior descending artery; ACC/AHA = American College of Cardiology/American Heart Association; DES = drug-eluting stent; atm = atmosphere; DCB = drug-coated balloon; IVL = intravascular lithotripsy; SC = semi-compliant; and NC = non-compliant.

**Table 3:** Peri- and post-procedural outcomes.

	*N* of Patients = 16
Pre-discharge ECG modifications, *n* (%)	0 (0)
Post-procedural type 4a MI, *n* (%)	0 (0) [12]
In-hospital MACE, *n* (%)	0 (0)
Cardiac death, *n* (%)	0 (0)
TV-MI, *n* (%)	0 (0)
TIA/Stroke, *n* (%)	0 (0)
Coronary perforation, *n* (%)	0 (0)
Flow-limiting dissection, *n* (%)	0 (0)
Abrupt vessel closure, *n* (%)	0 (0)
Sustained VT, *n* (%)	0 (0)

Data are number (*n* = number; % = percentage), as appropriate. ECG = electrocardiogram; MI = myocardial infarction; MACE = major adverse cardiovascular events; TV-MI = target vessel MI; TIA = transient ischemic attack; and VT = ventricular tachycardia.

**Table 4:** QCA.

	*N* of Patients = 16
Initial MLD, mm [IQR]	1.21 [1.06]
Distal reference diameter (RVD), mm [IQR]	2.5 [0.46]
Initial diameter stenosis, % [IQR]	89.5 [17]
Lesion length, mm [IQR]	28.9 [11.8]
Post-ShockFast™ MLD, mm [IQR]	2.1 [0.64]
Post-ShockFast™ residual diameter stenosis, % [IQR]	60.5 [32]
Final MLD, mm [IQR]	3.1 [0.53]
Acute lumen gain, mm [IQR]	1.86 [0.62]
Final diameter stenosis, % [IQR]	11.5 [9]

Data are median (IQR = interquartile range), or number (*n* = number; % = percentage), as appropriate. QCA = quantitative coronary analysis; MLD = minimal lumen diameter; and RVD = reference vessel diameter.

**Table 5:** QCA stratified by method of assessment (angiography-only vs. OCT-based).

	Angiography-Only = 8 Patients	OCT-Based = 8 Patients	*p*-Value
Initial MLD, mm (±SD)	1.27 ± 0.64	0.96 ± 0.64	0.351
Post-IVL MLD, mm (±SD)	2.14 ± 0.33	1.81 ± 0.58	0.177
Final MLD, mm (±SD)	3.01 ± 0.34	3.12 ± 0.57	0.645
Final Acute Lumen Gain, mm (±SD)	1.74 ± 0.60	2.15 ± 0.47	0.142
Initial DS, % (±SD)	80.25 ± 15.97	90.25 ± 7.38	0.130
Post-IVL Residual DS, % (±SD)	41.88 ± 20.92	66.25 ± 14.08	0.016
Final DS, % (±SD)	13.75 ± 5.92	12.5 ± 8.98	0.747
Lesion Length, mm (±SD)	28.86 ± 10.84	31.87 ± 18.76	0.701

Data are mean (SD = standard deviation), or number (*n* = number; % = percentage), as appropriate. QCA = quantitative coronary analysis; MLD = minimal lumen diameter; DS = diameter stenosis; OCT = optical coherence tomography; and IVL = intravascular lithotripsy.

**Table 6:** OCT characteristics.

	*N* of Patients = 8
Initial MLA, mm^2^ [IQR]	3.1 [1.6]
Initial MLD, mm [IQR]	1.94 [0.86]
Proximal reference diameter, mm [IQR]	3.5 [0.90]
Proximal EEL reference diameter, mm [IQR]	4.36 [0.41]
Distal reference diameter, mm [IQR]	2.5 [0.36]
Distal EEL reference diameter, mm [IQR]	3.27 [0.47]
Lesion length, mm [IQR]	41.5 [28.2]
Calcified nodule, *n* (%)	3 (38)
Eruptive calcified nodule, *n* (%)	3 (100)
Calcium arc, degrees [IQR]	274 [90]
Calcium thickness, mm [IQR]	1.75 [0.29]
Calcium longitudinal length, mm [IQR]	25.4 [21]
Revised OCT calcium score, *n* [IQR]	3 [0]
Original OCT calcium score, *n* [IQR]	4 [0]
Presence of calcium fractures post-IVL, *n* (%)	8 (100)
Calcium fractures > 2, *n* (%)	5 (63)
Post-IVL MLA, mm^2^ [IQR]	4.14 [3]
Final MSA, mm^2^ [IQR]	4.97 [3.53]
Final acute lumen gain, mm^2^ [IQR]	1 [2.85]
Final stent expansion, % [IQR]	90 [9]
Malapposition, *n* (%)	3 (38)
Major malapposition, *n* (%)	0 (0)
Medial edge dissection, *n* (%)	0 (0)

Data are median (IQR = interquartile range), or number (*n* = number; % = percentage), as appropriate. OCT = optical coherence tomography; MLA = minimal lumen area; MLD = minimal lumen diameter; EEL = external elastic lamina; and MSA = minimal stent area.

**Table 7:** Pre- and post-procedural cardiac biomarkers.

	N of Patients = 16
Initial Troponin, ng/L [IQR]	19 [15]
Initial CK, U/L [IQR]	76 [43]
Initial CK-MB, U/L [IQR]	16 [6]
Pre-discharge Troponin, ng/L [IQR]	35 [201]
Pre-discharge CK, U/L [IQR]	97.5 [41]
Pre-discharge CK-MB, U/L [IQR]	15 [9]

Data are median (IQR = interquartile range), as appropriate. CK = creatine kinase; CK-MB = creatine kinase MB.

## Data Availability

The raw data supporting the conclusions of this article may be made available by the authors on request.
